# A Holistic Approach to Evaluating Linear and Non-Linear Mixed Models to Predict Phosphorus Retention in Growing and Finishing Pigs

**DOI:** 10.3390/ani12131611

**Published:** 2022-06-22

**Authors:** Christopher D. Powell, Jennifer L. Ellis, Raquel S. Dias, Secundino López, James France

**Affiliations:** 1 Department of Animal Biosciences, University of Guelph, Guelph, ON N1G 2W1, Canada; jellis@uoguelph.ca (J.L.E.); raquelsdbetini@gmail.com (R.S.D.); jfrance@uoguelph.ca (J.F.); 2 Trouw Nutrition, Puslinch, ON N0B 2J0, Canada; 3 Departamento de Producción Animal, Universidad de León, 24007 León, Spain; s.lopez@unileon.es; 4 Instituto de Ganadería de Montaña, CSIC-Universidad de León, Finca Marzanas s/n, 24346 Grulleros, Spain

**Keywords:** phosphorus retention, growing and finishing pigs, non-linear models, endogenous phosphorus losses, phosphorus requirement for maintenance

## Abstract

**Simple Summary:**

Phosphorus (P) is an essential mineral in the diets of pigs. The degree to which P is utilized has both economic and environmental consequences to the swine industry. Mathematical models can be used to describe the relationship between P intake and P retention. These models provide information regarding P utilization that can be used to formulate diets aimed at reducing P overfeeding, and therefore decrease P wastage. The objective of this study was to assess the ability of four non-linear models (monomolecular, Michaelis-Menten, Richards, and Morgan) and one simple linear model to describe the relationship between P intake and P retention in growing and finishing pigs. Through fitting these models to data from P balance studies, non-linear models which describe diminishing returns type behaviour, the monomolecular and the Michaelis-Menten models, were found to best describe the relationship between P intake and P retention in these categories of pig. The parameter of these models allows estimates of endogenous P losses, P requirement for maintenance and theoretical maximum P retention enabling the more efficient use of P in the swine industry.

**Abstract:**

The ability of four non-linear mixed models and one linear mixed model to describe phosphorus (P) retention as a function of dietary P intake, expressed on an available P (avP) basis, was assessed in growing and finishing pigs. Of the four non-linear models, the monomolecular and Michaelis-Menten describe diminishing returns behaviour, while the Richards and Morgan describe sigmoidal behaviour with the ability to also describe diminishing returns. Using a meta-analysis approach, models were fitted to avP intake vs. P retention data from P balance studies. Pig bodyweights (BW) ranged from 43.5 to 133 kg, P intake ranged from 0.055 to 0.468 g kg^−1^ BW^0.75^ d^−1^ for avP, and 0.151 to 0.806 g kg^−1^ BW^0.75^ d^−1^ for total P, with P retention ranging from 0.026 to 0.329 g kg^−1^ BW^0.75^ d^−1^. Models were evaluated using statistical measures of goodness-of-fit and inspection of residuals. The monomolecular and Michaelis-Menten best described the relationship between P retention and P intake. Endogenous P losses and P requirement for maintenance were found to be higher in finishing pigs compared to growing pigs as BW increased.

## 1. Introduction

Non-ruminants have a limited ability to hydrolyze phytate into phosphate, the metabolically available form of phosphorus (P) that can be absorbed in the intestine. As a consequence of low phytate utilization, considerable levels of unabsorbed P are excreted in faeces. This not only represents a significant economic loss, but also an environmental pollutant compromising the sustainability of the swine industry [1,2,3]. In addition, although inorganic P sources are better utilized by the animal, these are finite resources and are prone to volatile prices [4,5,6]. Due to these concerns, a considerable number of studies on P utilization in pigs has been carried out, resulting in a significant body of data being available that can be used to improve understanding of the utilization of this mineral by pigs.

The fitting of mathematical functions to data from animal experiments has been shown to be useful in characterizing biological behaviour [7]. In a meta-analytic study, Schulin-Zeuthen et al. [8] reported that the monomolecular equation was the best estimator of P retention in growing pigs. Dilger and Adeola [9] also observed non-linear behaviour in the relationship between dietary P levels and P excretion in growing pigs fed conventional and low-phytate soybean diets. They pointed out the necessity for more studies to evaluate whether a linear relationship between P intake and P output would be the best functional form to estimate true P digestibility and endogenous P loss in pigs. In this work, four non-linear functions were assessed for their ability to describe the relationship between dietary P levels and P retention. Non-linear models include the monomolecular and the Michaelis-Menten, which describe diminishing returns behaviour, while the Richards and Morgan describe sigmoidal behaviour with the ability to also describe diminishing returns. Additionally, a simple linear model was applied.

As nutrient requirements vary with physiological state [10,11], in addition to relative rates of mineral deposition being influenced by body weight [12], it is crucial to understand P utilization in different categories of pigs to formulate diets to reduce P overfeeding and consequently P waste. Thus, the objective of this study was to evaluate linear and non-linear models in their ability to describe P utilization in growing and finishing pigs through the holistic approach of meta-analysis using data collected from various phosphorous balance studies.

## 2. Materials and Methods

### 2.1. Data Collection

A database was created using studies providing P balance data on growing and finishing pigs. The literature search was conducted using the University of Guelph provided Omni academic search engine. Key words included: “growing-finishing pigs”, “phosphorus balance” and “phosphorus retention”; search year ranged from 1966 to 2020. Using these criteria, 312 results were generated. These were narrowed down to a potential 106 papers based on excluding classes of pigs that were not growing and finishing i.e., nursing, weaning, lactating, etc. Only studies containing information on diet, total P (tP) dietary intake, body weight (BW), P excreted in faeces and urine, and P retention were selected. The inclusion of available P (avP) data allows for a more realistic analysis of assembled experiments with diets composed of distinct ingredients. Apparent total tract digestibility (ATTD) can serve as a method to estimate avP [13]. Therefore, in studies whereby avP was not explicitly reported, ATTD values of P were used. If neither avP nor ATTD values were reported, avP was calculated using reported tP and associated bioavailability of phosphorus values from the Nutrient Requirements of Swine (1998). Only treatments that did not include additional phytase supplementation were included in the database. In brief, the database contained 99 treatments from 23 studies encompassing 453 pigs with BW ranging from 43.5 kg to 133 kg, with an average BW across the entire database of 78.7 kg. P intake, in addition to P retention, was scaled by metabolic body weight (g kg^−1^ BW^0.75^ d^−1^). Across the database intake of avP ranged from 0.055 to 0.468 g kg^−1^ BW^0.75^ d^−1^, tP from 0.151 to 0.806 g kg^−1^ BW^0.75^ d^−1^ with P retention ranging from 0.026 to 0.329 g kg^−1^ BW^0.75^ d^−1^. Table 1 contains a summary of the averaged values, and their range, contained in the full dataset, in addition to the sex. Please refer to Table A1 in the Appendix A, for body weight, breed, and dietary description of the individual studies encompassing the full dataset. The full dataset can be found in the associated Supplementary Material section of this article.

### 2.2. Matheamtical Considerations

Five functional forms were used to describe the profiles resulting from the relationship between tP, avP and P retention, viz.

(1) Straightline:y=cx−b

(2) $$\mathrm{Monomolecular}:y=a-\left(a+b\right)e^{-cx}$$

(3) $$\mathrm{Michaelis}-\mathrm{Menten}:y=\frac{-bc+ax}{c+x}$$

(4) $$\mathrm{Richards}:y=\frac{b\left(a+2b\right)}{{\left\{b^{n}+\left[{\left(a+2b\right)}^{n}-b^{n}\right]e^{-cx}\right\}}^{\frac{1}{n}}}-2b\;$$

(5) $$\mathrm{Morgan}:y=\frac{-bc^{n}+ax^{n}}{c^{n}+x^{n}}$$

where variable *x* denotes P intake (tP or avP) and *y* denotes P retention, with both of these variables being expressed on a g kg^−1^ BW^0.75^ d^−1^ basis. Parameter *a* represents theoretical maximum retention, while parameter *b* represents endogenous P excretion. In the non-linear equations, parameters *c* and *n* define the shape of the curve, while *c* represents the slope in the linear equation. Parameters are all positive except *n* ≥ −1 for the Richards. The above equations are modifications of their standard forms, because equations for describing P balance data require a negative intercept on the ordinate axis. The standard form of the Michaelis-Menten intercepts the *y*-axis at the origin and standard growth functions (e.g., monomolecular, Richards and Morgan) give a positive intercept (refer to Thornley and France [7] for details). Equations (2) and (3) describe diminishing returns behaviour, whilst Equations (4) and (5) are also capable of describing sigmoidal behaviour with a variable point of inflexion. Equation (2) is a special case of Equation (4) (i.e., *n* = −1), and Equation (3) is a special case of Equation (5) (i.e., *n* = 1).

For each function, P requirement for maintenance was calculated by setting *y* equal to zero and solving for *x*, viz.

(6) Straightline:x=b/c

(7) $$\mathrm{Monomolecular}:x=c^{-1}\ln\left[\left(a+b\right)/a\right]$$

(8) $$\mathrm{Michaelis}-\mathrm{Menten}:x=c\frac{b}{a}$$

(9) $$\mathrm{Richards}:x=c^{-1}\ln\left\{\frac{2^{n}\left[{\left(a+2b\right)}^{n}-b^{n}\right]}{{\left(a+2b\right)}^{n}-{\left(2b\right)}^{n}}\right\}$$

(10) Morgan:x=c(ba)1n

Additionally, following the methods of Darmani Kuhi et al. [14], the change in retention based upon the difference between two P intakes, expressed as retention efficiency ($\bar{k}$), was calculated:

(11) $$\bar{k}=\frac{\Delta y}{\Delta x}\;$$

### 2.3. Statistical Analysis

#### 2.3.1. Model Development

Initially, each function was fitted using the PROC NLIN procedure in SAS [15] which does not consider random effects. This procedure was used to assist in determining initial parameter estimates for each model. These initial estimates were then used to perform a regression analysis using a meta-analytic approach that considered each study as a random effect [16]. The PROC NLMIXED procedure of SAS (2000) was used for this analysis (PROC MIXED for linear functions). The distribution of random effects was assumed to be normal. The dual quasi-Newton technique was used for optimization with adaptive Gaussian quadrature as the integration method. Using the PROC SURVEYSELECT, the BALBOOTSTRAP method was used for balanced bootstrap sampling of the original dataset. The same seed was used between models to ensure the resulting bootstrapped database to which the models were fitted was the same. Using this method, the sample size for each bootstrap replicate is equal to the original sample size with equal sampling probability and replacement [17]. One thousand (1000) bootstrapping replicates were specified.

#### 2.3.2. Model Evaluation

Models were evaluated using common statistical measures of goodness-of-fit in addition to inspection of residuals. Agreement between model predictions and observed values was assessed using mean square prediction error (MSPE):

$$\mathrm{MSPE}=\frac{1}{n}\overset{n}{\underset{i=1}{\sum }}{\left(O_{i}-P_{i}\right)}^{2}$$

where *n* is the number of observations, *O_i_* is the observed value, and *P_i_* is the predicted value [18]. The MSPE was decomposed into errors in central tendency (*u*^M^), errors due to regression (*u*^R^), and errors due to disturbance (*u*^D^) [19]. Additionally, agreement between model predictions and observations was tested using the concordance correlation coefficient (CCC). Following the methods proposed by Lin [20], calculated CCC values range from −1, representing perfect disagreement, to +1, perfect agreement, with a value of 0 indicating no agreement between observed and predicted values. The Akaike information criterion (AIC) was used to compare models against one another [21]. The AIC is used for model selection whereby both the goodness-of-fit and the number of parameters in that model are accounted for. This criterion penalizes more complicated models for inclusion of additional parameters; using these criteria models with lower AIC values are preferred.

## 3. Results

The ability of four non-linear and one linear model viz. monomolecular, Michaelis-Menten, Richards, Morgan, and linear, to describe phosphorus retention in growing and finishing pigs given avP intake, expressed on a metabolic BW basis, was assessed using a meta-analytic approach. Due to the nature of their derivation, the models yielded biologically meaningful parameters including theoretical maximum retention (*a*) and endogenous P excretion (*b*), in addition to allowing for the calculation of P requirement for maintenance based upon the parameter estimates.

### 3.1. Fitting Behaviour

Of the four non-linear models, two described diminishing returns behaviour alone, viz. the monomolecular, Michaelis-Menten, while the remaining two described sigmoidal and diminishing returns behaviour, the Richards and Morgan. When fitting the linear, monomolecular and Michaelis-Menten models to either the avP intake dataset, no convergence or fitting issues were encountered. When fitting the Richards equation to the avP intake, instead of describing a sigmoidal type response, the Richards reverted to the monomolecular (i.e., *n* = −1) and thus described a diminishing returns response. Likewise, the Morgan describes a sigmoidal response, and although it did not revert to the Michaelis-Menten (i.e., *n* ≠ 1) when fitted to either the avP, the shape parameter *n* did approach 1, viz. 2.16, with values of *n* closer to 1 resulting in a more diminishing returns style of behaviour. When fitting both the Richards and Morgan, the parameter *b*, endogenous P excretion, tended to converge on a negative number. As *b* must be greater than 0 from a biological basis, a bound forcing *b* to be greater than 0 was implemented when fitting these models. However, as seen in Table 2, for both these models the parameter *b* converged to very small positive numbers.

### 3.2. Parameter Estimates, Derived Parametersand Fitted Values

**Figure 1:**
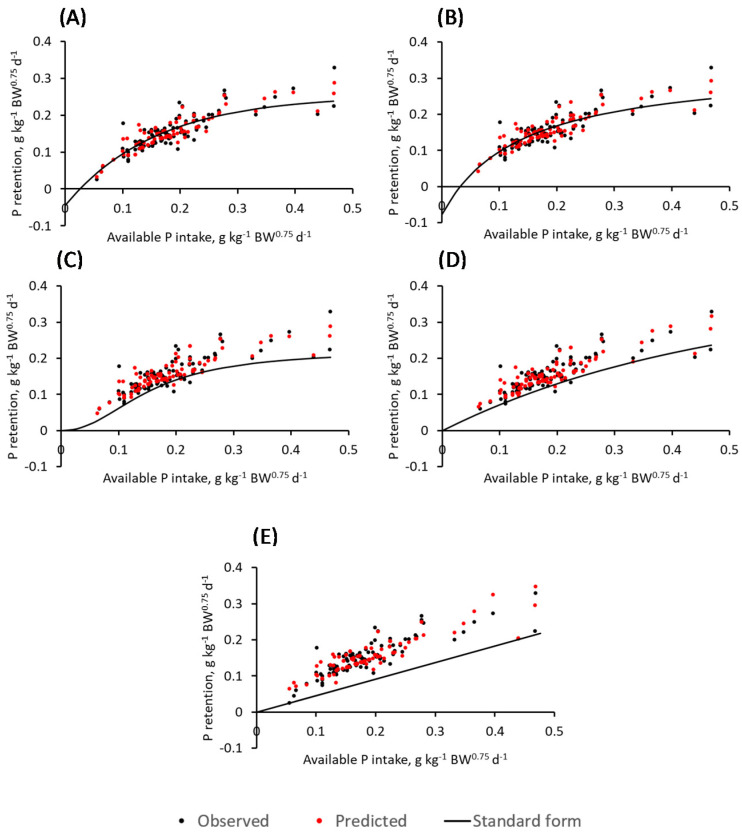
Observed and predicted values resulting from fitting the monomolecular (**A**), Michaelis-Menten (**B**), Morgan (**C**), Richards (**D**) and linear (**E**) to the available P intake dataset. Predicted values represented by the red markers include the random effect of study while the black solid line represents predicted values of the standard equation forms, viz. Equations (1)–(5) using the parameter estimates from Table 2.

Parameter estimates, in addition to their 95% confidence intervals, resulting from fitting the linear and four non-linear models to avP intake to describe P retention are presented in Table 2. Comparing parameters with biological significance, values of *a* (theoretical maximum P retention) were 0.22, 0.25, 0.34, and 0.37 g kg^−1^ BW^0.75^ d^−1^ for the Morgan, monomolecular, Michaelis-Menten and Richards, respectively, when fitted to the P retention vs. avP intake. Given the same avP dataset, endogenous P excretion (*b*) values for the monomolecular and Michaelis-Menten were 0.04 and 0.08 g kg^−1^ BW^0.75^ d^−1^, respectively. For the linear and Morgan, parameter estimates representing endogenous P excretion were 1.0 × 10^−8^ g kg^−1^ BW^0.75^ d^−1^ while for the Richards 3.0 × 10^−16^. Figure 1 displays the predicted values of retained P of all five models, represented by red markers, vs. the observed values, represented by black markers, when fitted to the avP intake data. The predicted values represented by the red dots include the random effect of study in the model while the continuous black line represents predicted values using the standard form of the equation.

P requirement for maintenance calculated using Equations (6)–(10) was 0.023, 0.024 and 0.033 g kg^−1^ BW^0.75^ d^−1^ for the monomolecular, Richards and Michaelis-Menten, respectively. The Morgan and linear models resulted in very low estimates for P requirement for maintenance, 6.0 × 10^−5^ and 2.2 × 10^−8^ g kg^−1^ BW^0.75^ d^−1^, respectively.

Phosphorus retention efficiency,$\;\bar{k}$, over various ranges of P intake is presented in Table 2. For all non-linear models, when calculating phosphorus retention efficiency based upon various ranges of P intake, over the observed avP intake rage, viz. 0.055 to 0.468 g kg^−1^ BW^0.75^ d^−1^, higher efficiencies were seen in the lower half of the P intake range (0.055 to 0.207) compared to the upper half (0.207 to 0.468). In the linear model, phosphorus retention efficiency is represented by the slope, 0.46 g kg^−1^ BW^0.75^ d^–1,^ and thus is constant regardless of P intake level.

### 3.3. Model Evaluation

**Figure 2:**
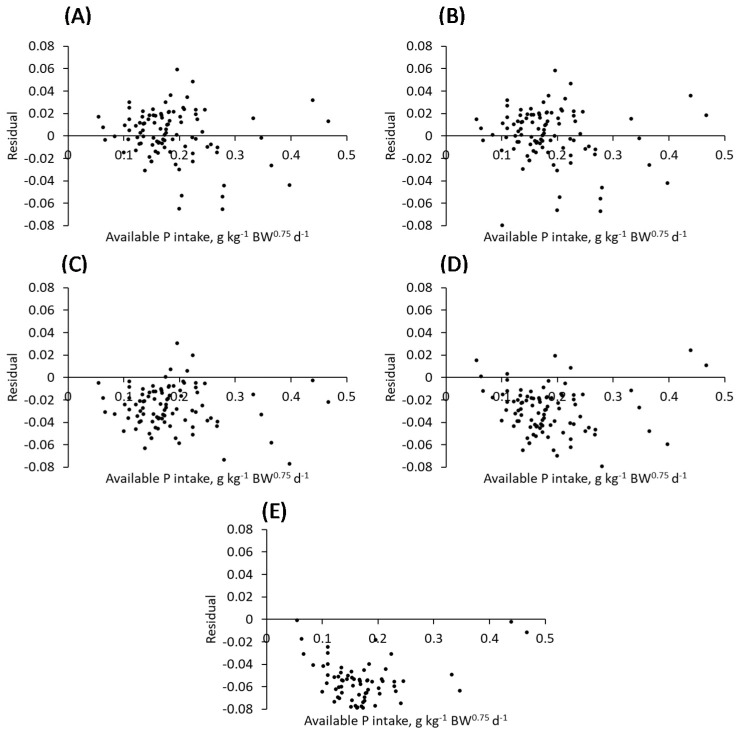
Plotted residuals of retained P for the monomolecular (**A**), Michaelis-Menten (**B**), Morgan (**C**), Richards (**D**) and linear (**E**) when fitted to the available P intake dataset (g kg^−1^ BW^0.75^ d^−1^).

Models were evaluated based upon goodness-of-fit and model selection criteria in addition to inspection of residuals. Figure 2 displays the plotted residuals of all five models when predicting P retention based on avP intake using the standard form of the equations, i.e., not including the random effect of study. From visual inspection of these plots, no clear pattern in residuals is observed in panel (A) and (B) representing the monomolecular and the Michaelis-Menten, respectively. In contrast, panels (C) to (E), representing the Morgan, Richards and linear, display a clear pattern of consistently negative residuals. The resulting goodness-of-fit criteria, viz. CCC, MSPE and its decomposition for individual models when fitted to avP intake data are displayed in Table 3, in addition to AIC values. Models were evaluated as fixed effect models and as mixed models whereby both fixed and the random effect of study were taken into consideration. Non-linear models fitted to the avP intake data resulted in higher CCC values and lower MSPE values, indicating superior fits in comparison to the linear model. Likewise, AIC values were smaller when comparing the non-linear and linear models. When evaluating the fixed effect models, those which describe diminishing returns, the monomolecular and Michaelis-Menten, had the highest CCC values of 0.821 and 0.842, respectively. The Morgan and Richards had higher CCC values compared to the linear, 0.663, 0.645 and 0.362, respectively, but less than the monomolecular and Michaelis-Menten. AIC was lowest for the Michaelis-Menten (−723.5), followed by the monomolecular (−723.0), Morgan (−630.7), Richards (−619.1) and linear (−513.9). MSPE followed the same trend as AIC, with MSPE lowest for the Michaelis-Menten and monomolecular and highest for the linear. From the MSPE decomposition, the vast majority of the error (+96%) was in *u*^D^, errors due to disturbances, for the monomolecular and Michaelis-Menten. In contrast to the linear, Richards and Morgan prediction error accumulated in errors in the central tendency term (*u*^M^), indicating high overall bias in model predictions.

On the basis of AIC, CCC and MSPE, all models performed better using a mixed model approach and incorporating the random effect of study compared to a fixed effect model. In agreement with the fixed effect methodology, the monomolecular and Michaelis-Menten preformed superior in comparison to the Morgan, Richards and linear. When comparing MSPE values in a given model between the fixed effect and mixed approach, a greater decrease in MSPE is observed in the poorer fitting models, viz. linear Richards and Morgan. This means that unexplained variance which the fixed effect parameter cannot explain is being attributed to the random effect in the mixed model. However, there is a smaller difference between MSPE values for the monomolecular and Michaelis-Menten between the fixed and mixed models. Therefore, the parameters in the fixed effect models are able to capture the major factors describing the relationship between avP intake and P retention.

## 4. Discussion

Nutrient requirements in addition to P utilization by pigs vary based upon physiological status and weight [10,11]. Additionally, both linear and non-linear models have been applied to describe P responses in pigs, including digestibility, retention, and exogenous excretions, when fed various levels of dietary P [8,22,23,24,25]. Given this, the objective of the present study was to access the ability of various non-linear and linear models to describe P retention data in growing and finishing pigs in response to avP intake.

### 4.1. Candidate Functions and Response Shape of P Retention

In total, five functions were fitted to a dataset encompassing P retention in growing and finishing pigs in response to varying P intake levels, expressed on a total or available basis. These functions can be broadly categorized based upon the response shape that they describe. Of the five functions, one describes a linear response while the other four cover non-linear responses. The four non-linear functions can be categorized based upon their ability to describe diminishing returns style responses, viz. the monomolecular and the Michaelis-Menten, and/or sigmoidal style responses, viz. the Richards and Morgan. Diminishing returns behaviour is characterized by the rate of change in the independent variable, in this case change in P retention in response to change in P intake (dy/dx), being initially maximal and continuously decreasing with increasing P intake as the upper asymptote is approached. In contrast, sigmoidal behaviour is characterized by an initially increasing rate of change, represented for example by an exponential, until a maximal rate of change is achieved at the inflexion point, thereafter the rate of change is continuously decreasing in approaching the upper asymptote. With a simple linear function, the rate of change is constant, as represented by the slope parameter.

Based upon goodness-of-fit criteria, non-linear diminishing returns type functions, viz. the monomolecular and the Michaelis-Menten, best described P retention in growing and finishing pigs in response to P intake, expressed on an available basis. This was further reinforced by the fact that the Richards, which has the ability to describe both sigmoidal and diminishing type behaviour, reverted to the monomolecular, viz. *n* = −1, and thus described a diminishing returns behaviour pattern when fitted to the avP intake data. The linear function was found to fit the data the poorest compared to the non-linear functions when fitted to the avP dataset. This is not surprising as non-linear behaviour of P utilization in pigs has been demonstrated previously by Dilger and Adeola [9], Kebreab et al. [26], Létourneau-Montminy et al. [27] and Schulin-Zeuthen et al. [8] among others. A possible explanation of why certain studies observed linear response trends is the potential narrow range in which dietary P intake was provided in a given study. Biological responses are rarely linear, with non-linearity becoming prevalent at the upper range of doses or intakes [28]. In the study of Schulin-Zeuthen et al. [8], when fitting non-linear models to P balance data in growing pigs, the authors suggested that the parameter representing the upper asymptote of their models was not well fitted due to the lack of sufficient data above 0.4 g avP kg^−1^ BW^0.75^ d^−1^. In contrast, the current study encompasses data whereby intakes approached 0.5 g avP kg^−1^ BW^0.75^ d^−1^ resulting in a much better-defined upper asymptote. Furthermore, in the study of Pettey et al. [24] the authors determined a linear response of P retention in three P balance studies of pigs weighing 27, 59 and 98 kg. However, in the largest weight class of pigs, P intake when expressed on a metabolic BW basis was between 0.06 and 0.20 g kg^−1^ BW^0.75^ d^−1^. If findings of the study of Pettey et al. [24] were extrapolated to P intakes outside of the study’s range, caution should be taken as it is reasonable to conclude that the linearity observed may become non-linear as P intake continues to increase to values outside of the observed range of the study.

### 4.2. Endogenous P Excretion, Requirement for Maintenance, and Efficiency

In the current study, endogenous P excretion, represented by the parameter *b,* varied based upon the model fitted. When fitting to the avP intake data, the Michaelis-Menten resulted in the value of 0.08 g kg^−1^ BW^0.75^ d^−1^ compared to 0.04 g kg^−1^ BW^0.75^ d^−1^ using the monomolecular. Regardless of the model applied, endogenous P excretion in the current study was greater than that of studies conducted on growing pigs [8,9,24,25,29]. Using a similar meta-analytic approach, Schulin-Zeuthen et al. [8] determined endogenous P excretion in growing pigs with an average body weight of 59.2 kg to be 0.014 g kg^−1^ BW^0.75^ d^−1^ when fitting the monomolecular to P retention vs. avP intake data. From 66 P balance studies with pigs ranging from 30 to 70 kg, Rodehutscord et al. [29] determined endogenous P excretion to be 0.015 g kg^−1^ BW^0.75^ d^−1^ using regression methodology. Furthermore, Rodehutscord et al. [29] suggested that endogenous P losses are dependent on body weight, which is in agreement with the current study.

P requirement for maintenance was determined by solving for the P intake value when P retention was zero for each of the applied models. Limited data exist regarding P requirements for maintenance as P requirements for pigs are most commonly expressed on a total basis whereby both obligatory losses and P retention are summed [8,30]. In the study of Schulin-Zeuthen et al. [8] the authors concluded P requirement for maintenance was 0.015 g kg^−1^ BW^0.75^ d^−1^ when fitting the monomolecular to pigs ranging from 20 to 99 kg with an average body weight of 59 kg. Comparatively, P requirement for maintenance in larger pigs (average body weight of 79 kg) in the current study resulted in a higher value, 0.023 g kg^−1^ BW^0.75^ d^−1^, using the monomolecular model.

The average efficiency of converting P intake on a total and available basis to retained P was calculated for each individually fitted model. Average efficiency was calculated in three ways; from minimum to the maximum P intake value (0.055 to 0.468 g avP kg^−1^ BW^0.75^ d^−1^), from 0.055 to the mid-point (0.055 to 0.207 g avP kg^−1^ BW^0.75^ d^−1^) and from the mid-point to the maximum (0.207 to 0.468 g avP kg^−1^ BW^0.75^ d^−1^). Examining the monomolecular, the average efficiency of converting dietary P intake into retained P was substantially higher in the lower half of the P intake range compared to the upper half, viz. 0.83 vs. 0.24, with an overall average efficiency of 0.46. Likewise, in the study of Schulin-Zeuthen et al. [8] P retention efficiency was higher in the first half (0.75) of the P intake range compared with the second (0.54) when applying a non-linear model, viz. the monomolecular. A direct comparison between the efficiencies as reported in the current study and those of that of Schulin-Zeuthen et al. [8] is not entirely apt. The very mathematical nature of an equation which describes diminishing returns behaviour, such as the monomolecular and Michaelis-Menten, describes a scenario whereby efficiency of converting P intake into P retention (dy/dx) starts at a maximum and continuously decreases towards zero as the upper asymptote is approached, i.e., with increasing P intake the efficiency of retention decreases. In the study by Schulin-Zeuthen et al. [8], the avP intake ranged from 0.1 to 0.5 g kg^−1^ BW^0.75^ d^−1^, with the authors suggesting that the upper asymptote of their fitted monomolecular model was not well defined due to lack of observations above 0.4 g kg^−1^ BW^0.75^ d^−1^. Therefore, if the range of avP intake data used in the study does not result in the upper asymptote of P retention been approached, the rate at which P intake is converted into retained P can be decreased further by a not insignificant amount. However, the relative avP intake range, when expressed on a metabolic body weight basis, between the present study and that of by Schulin-Zeuthen et al. [8] is comparable, 0.055 to 0.468 and 0.10 to 0.50 g kg^−1^ BW^0.75^ d^−1^, respectively. In this range of intakes the growing pigs (BW 20–90 kg) of Schulin-Zeuthen et al. [8] displayed a higher efficiency of converting avP intake into retained P (0.65) compared to the current study on heavier pigs (BW 43.5 to 133.0 kg) with a efficiency of 0.46. These findings are in agreement with those of Ruiz-Ascacibar et al. [12], whereby relative mineral deposition rate, including P, decreased as body weights increased in intact males, castrates and female pigs from 40 to 140 kg.

In contrast to non-linear models, in a simple linear model the efficiency of converting P intake into P retention (the slope) is constant regardless of the P intake value. In the current study, parameter *c,* the slope which represents the efficiency of converting dietary P into retained P was 0.46, meaning that for every 1 g increase in avP intake 0.46 g would be retained on a g kg^−1^ BW^0.75^ d^−1^ basis. This relationship holds no matter what the dietary P intake value is. The ramifications of this assumption are apparent when comparisons are made between the linear and non-linear models. Increasing the avP intake value from 0.1 to 0.2 and from 0.4 to 0.5 g kg^−1^ BW^0.75^ d^−1^ using the linear model, would cause P retention to increase by 0.046 g kg^−1^ BW^0.75^ d^−1^ over either range. In contrast, using the monomolecular, increasing the avP intake value from 0.1 to 0.2 g kg^−1^ BW^0.75^ d^−1^ causes P retention to increase from 0.096 to 0.168 g kg^−1^ BW^0.75^ d^−1^; an increase of 0.072 g kg^−1^ BW^0.75^ d^−1^ and an efficiency of 0.72 over this 0.1 increase in avP. When dietary avP intake increases from 0.4 to 0.5 g kg^−1^ BW^0.75^ d^−1^ P retention increases from 0.227 to 0.238 g kg^−1^ BW^0.75^ d^−1^, an increase of 0.011 g kg^−1^ BW^0.75^ d^−1^ and efficiency of 0.11, over this 0.1 increase in avP. Therefore, if one was to apply the linear model rather than the non-linear monomolecular model to this dataset, the linear model would under-predict the efficiency of converting dietary P into retained P at lower levels of P intake and grossly over-predict the efficiency at higher intake levels. The decrease in efficiency in converting dietary P into retained P as dietary P intake increases makes sense from a biological standpoint, i.e., there is greater utilization efficiency when P intake is below P requirement and a subsequent decrease as this requirement is met [31]. The non-linear behaviour of the relationship between avP intake and P retention suggests that determination of endogenous P loss and true digestibility is better undertaken using non-linear models rather than the commonly used linear regression analysis [23,24].

## 5. Conclusions

Using a meta-analytic approach, a database was created that described P retention in response to dietary available P intake in growing and finishing pigs. The response of P retention to dietary avP intake was best described using simple non-linear models that describe patterns of diminishing returns behaviour, viz. the monomolecular and Michaelis-Menten. Furthermore, endogenous P losses and P requirements for maintenance were found to be higher as BW increased, whilst the efficiency of converting dietary P intake into retained P was reduced.

## Figures and Tables

**Table 1:** Averages and minimum-maximum values of bodyweight (BW, kg), P intake and P retention values of the dataset in addition to being expressed on the basis of sex.

Item	Full Dataset	Sex	
		Male	Female
*n*	99	80	19
BW, kg	78.7	80.5	71.4
	43.5–133.0	48.4–133.0	43.5–104.1
P measurements, g d^−1^			
tP intake	11.3	11.2	11.6
	3.7–21.7	4.3–20.0	3.7–21.7
avP intake	4.8	4.6	5.8
	1.5–13.0	1.6–13.0	1.5–12.2
P retention	4.0	3.9	4.5
	0.7–8.8	0.7–8.8	1.4–8.6
P measurements, g kg^−1^ BW^0.75^ d^−1^			
tP intake	0.437	0.427	0.471
	0.151–0.806	0.151–0.785	0.162–0.806
avP intake	0.187	0.175	0.237
	0.055–0.468	0.055–0.467	0.066–0.468
P retention	0.153	0.146	0.185
	0.026–0.329	0.026–0.247	0.061–0.329

**Table 2:** Parameters estimates, and associated 95% confidence intervals, resulting from fitting linear and non-linear models to P retention vs. P intake (g kg–1 BW0.75 d–1) data in growing and finishing pigs, derived P requirement for maintenance in addition to average efficiency ($\bar{k}$ ) of dietary P conversation to retained P.

Item	Linear	Monomolecular	Michaelis-Menten	Richards	Morgan
Parameter ^1^					
*a* CI ^2^		0.25 0.24–0.26	0.34 0.33–0.35	0.37 0.37–0.38	0.22 0.22–0.22
*b* CI	1 × 10^−8^	0.044 0.04–0.05	0.08 0.07–0.09	3.0 × 10^−16^	1 × 10^−8^
*c* CI	0.46 0.45–0.46	6.33 6.27–6.40	0.14 0.14–0.14	2.12 2.12–2.13	0.15 0.15–0.16
*n* CI				−1.00 ^ᴪ^	2.16 2.12–2.21
Prequirement ^3^	2.2 × 10^−8^	0.023	0.033	0.024	6 × 10^−5^
${\bar{k}}_{\left(min-max\right)}\;$ ^4^	0.46	0.46	0.50	0.47	0.44
${\bar{k}}_{\left(min-mid\right)}\;$ ^5^		0.83	0.87	0.60	0.82
${\bar{k}}_{\left(mid-max\right)}\;$ ^6^		0.24	0.28	0.39	0.21

^1^ Parameter *a* represents the theoretical maximum P retention (g kg^−1^ BW^0.75^ d^−1^); parameter *b* represents endogenous P excretion (g kg^−1^ BW^0.75^ d^−1^); *c* and *n* are shape parameters; ^2^ 95% confidence limit of parameter estimate; ^3^ P requirement for maintenance (g kg^−1^ BW^0.75^ d^−1^); ^4,5,6^ where min, mid and max represent the minimum, middle and maximum observed avP intake values of the dataset, 0.055, 0.207 and 0.468 g kg^−1^ BW^0.75^ d^−1^, respectively; ^ᴪ^ n = ^−1^ therefore the Richards reverted to the monomolecular.

**Table 3:** Evaluation of models based upon goodness-of-fit and model selection criteria: concordance correlation coefficient (CCC), mean square prediction error (MSPE), its decomposition expressed as a percentage and Akaike information criterion (AIC), after fitting P retention vs. available P intake (g kg^−1^ BW^0.75^ d^−1^) data in growing and finishing pigs using both fixed and mixed methods.

Item	Linear	Monomolecular	Michaelis-Menten	Richards *	Morgan
Fixed effect					
CCC	0.362	0.821	0.842	0.645	0.663
MSPE	5.3 × 10^−3^	6.3 × 10^−4^	6.3 × 10^−4^	1.7 × 10^−3^	1.6 × 10^−3^
MSPE decomposition					
Errors in central tendency (*u*^M^)	86.0	0.0	0.0	63.6	58.8
Errors due to regression (*u*^R^)	0.6	2.86	3.4	1.3	0.9
Errors due to disturbances (*u*^D^)	13.4	97.1	96.6	35.1	40.3
AIC	−513.9	−723.0	−723.5	−619.1	−630.7
Fixed + random effects					
CCC	0.898	0.957	0.956	0.894	0.913
MSPE	4.3 × 10^−4^	2.6 × 10^−4^	2.1 × 10^−4^	2.8 × 10^−4^	3.5 × 10^−4^
MSPE decomposition					
Errors in central tendency (*u*^M^)	0.0	0.1	0.7	0.0	0.3
Errors due to regression (*u*^R^)	5.6	0.0	0.0	1.7	0.6
Errors due to disturbances (*u*^D^)	94.4	99.9	99.3	98.3	99.1
AIC	−762.8	−809.4	−830.2	−766.4	−779.0

* Richards reverted to the monomolecular when fitted to the avP intake data.

## Data Availability

The data presented in this study is available in the associated Supplementary Material of this article.

## Supplementary Materials

The following supporting information can be downloaded at: https://www.mdpi.com/article/10.3390/ani12131611/s1, Data used in this study can be found in the associated supplementary materials. Data pertaining to available phosphorus (avP) intake and phosphorus retention can be found in the Excel file entitled “Supplementary Material S1” while the dataset pertaining to the total phosphorus (tP) intake and phosphorus retention can be found in the Excel file entitled “Supplementary Material S2”.

## Appendix A

**Table A1:** Data description of studies generated from the literature in order to evaluate the ability of linear and non-linear models to describe phosphorus retention in growing and finishing pigs.

Study	Main Dietary Ingredients	Pig Breed	$\bar{BW}{\;}^{1},\mathrm{kg}$	References
1	High available P corn combined with high fat and protein corn diets	Crossbred	98	Hankins et al. [32]
2	Mutant corn hybrids	Crossbred	110	Hankins et al. [33]
3	Mainly corn with SBM	Crossbred	100	Hankins et al. [34]
4	Soybean meal and sorghum	DK97 and DK88 boars and DK33 and DK30 sows	99	O’Quinn et al. [35]
5	Barley, wheat, soybean meal combining different levels of P and Ca:P ratio	Large White × Landarace	61–64	Brady et al. [36]
6	Hulled Barley	Large White × Landarace	48	Htoo et al. [37]
7	Barley, SBM, Corn	German Large White × Pietran	62–88	Walz et al. [38]
8	Corn, barley, and various combinations of dietary fat and Ca levels	Pure Yorkshire	51–120	Jongbloed [39]
9	Corn, barley with soybean meal, and 4 levels of dietary fibre	Landrace × Yorkshire	62–95	Jongbloed [39]
10	Corn and barley with soybean meal	Landrace × Yorkshire	65–99	Jongbloed [39]
11	Corn and barley with soybean meal	Landrace × Yorkshire	73–99	Jongbloed [39]
12	Corn and barley with soybean meal	Niew Dalland × Landrace	54–98	Jongbloed [39]
13	Wet barley protein, wet distillers solids	Yorkshire × Landrace	90	Buraczewska et al. [40]
14	Corn, SBM	Yorkshire × Hampshire	90–91	Carter et al. [41]
15	Corn, barley, SBM	Unknown	54–60	Ekpe et al. [42]
16	Semi-purified	Yorkshire × Landrace	65	Fernández [43]
17	Typical diets found in DK, VN, TH	Unknown	57–60	Jørgensen et al. [44]
18	Semi-purified	Yorkshire × Landrace	59–98	Pettey et al. [24]
19	Corn SBM	Line 327 × C22	113	Hanni et al. [45]
20	Barley, wheat	Large White × (Large White × Landrace)	50	O’Doherty et al. [46]
21	Barley, SBM	Canabrid × Camborough	80	Sauer et al. [47]
22	Barley, wheat, SBM with three levels of P	CrossBred	44–104	Sørensen et al. [48]
23	Barley, corn	German Large White × German Land Race × Pietrain	90	Walz & Pallauf [49]

^1^ Average pig body weight per study, range of body weights indicate range of treatment means.
